# Ketogenic diets initiated in late mid-life improved measures of spatial memory in male mice

**DOI:** 10.1007/s11357-023-00769-7

**Published:** 2023-03-18

**Authors:** Zeyu Zhou, Kyoungmi Kim, Jon J. Ramsey, Jennifer M. Rutkowsky

**Affiliations:** 1 Department of Molecular Biosciences, School of Veterinary Medicine, University of California, Davis, CA USA; 2 Department of Public Health Sciences, School of Medicine, University of California, Davis, CA USA

**Keywords:** Ketogenic diet, Aging, Health span

## Abstract

Studies have shown ketogenic diets (KD) started from early middle-age improved health span and longevity in mice. KDs started later in life or administered intermittently may be more feasible and promote compliance. Therefore, this study sought to test if continuous or intermittent KDs started in late-middle-aged mice would improve cognition and motor function at advanced age. Eighteen-month-old male C57BL/6JN mice were assigned to an isocaloric control (CD), KD, or intermittent ketogenic (IKD, 3-day KD/week) diet. A panel of behavior tests were performed to assess cognitive and motor functions with aging. Y-maze alternation rate was higher for both IKD and KD mice at 23 months of age and for KD mice at 26 months indicating an improved spatial working memory. Twenty-six-month-old KD mice also showed better spatial learning memory in Barnes maze when compared to the CD. Improved grid wire hang performance was observed in aged IKD and KD versus CD mice indicating better muscle endurance under isometric contraction. A reduced level of circulating proinflammatory cytokines in aged KD (IL-6 and TNF-α) and IKD (IL-6) mice may contribute to the phenotypic improvements observed with these interventions. This study demonstrates that when initiated at late-middle age, the KD improved measures of spatial memory and grid wire hang performance in aged male mice, with IKD showing results intermediate to the CD and KD groups.

## Introduction

In recent years, there has been growing interest in the application of ketogenic diets (KD) to improve health span and manage age-related diseases, including Alzheimer’s disease, type 2 diabetes, Parkinson’s disease, and cancer [1–3]. KDs are depleted in dietary carbohydrate and high in fat and can mimic some of the metabolic changes with calorie restriction and fasting, including an increase in fatty acid oxidation and stimulation of endogenous ketogenesis [4]. An increased level of β-hydroxybutyrate, the main circulating ketone body, has also been associated with anti-aging phenotypes [3, 5–7] and signaling pathways [8, 9]. It has been shown that a KD started at 12 months of age extended longevity and improved motor function, as well as cognition, in aged mice [10], suggesting that KDs may show efficacy as interventions to promote healthy aging. In humans, long-term adherence to a strict dietary intervention is difficult, and interventions started later in life or intermittently may be more feasible and promote compliance.

Several studies have investigated the impact on aging of interventions initiated in late–middle-aged mice. It has been reported that dietary supplementation with α-ketoglutarate from 18 months of age increased lifespan in female mice and improved frailty in aged female and male mice [11]. Rapamycin feeding started at 19 months of age increased lifespan and motor coordination in mice [12]. A diet low in branched chain amino acids started at 16 months of age promoted metabolic health and robustness in aged mice [13]. In regard to a KD, rats fed a medium-chain triglyceride (MCT) supplemented KD from 20 months of age for 3 months showed improved performance in a cognitive dual task that involves both spatial working memory and bi-conditional association memory [14], although it is not known if other measures of health span would also be improved. These studies highlight the fact that aging interventions started in late-middle-age can mitigate age-related declines in physiological functions. Thus, one goal of the present study was to investigate if a KD initiated at 18 months of age would improve measures of cognition and motor function in old mice.

In addition to the effects of a KD started in late-middle-aged mice, the other arm of the study was to test if an intermittent KD (IKD), which consisted of 3 consecutive days of a KD each week, would produce similar health promoting effects as the continuous KD. Previous intermittent dietary interventions, including alternate day fasting [15, 16] and adherence to a 4-day fasting mimicking diet [17], have been reported to increase lifespan and improve some measures of health span at old age. These studies involved periods of decreased energy intake, and the present study investigated if intermittent periods of nutritional ketosis without a decrease in energy intake would induce some of the positive effects as seen in these studies. Regarding intermittent interventions of a KD, a weekly cyclic KD was reported to reduce midlife mortality and improve health span in aged mice [18] even though the mice during the KD cycles had increased caloric intake and weight gain. The present study paid particular attention to a shorter 3-day KD paradigm, and mice were fed an isocaloric amount of diet daily to maintain a relatively stable middle-age body weight throughout the study.

This study sought to investigate whether a continuous KD or a 3-day IKD initiated at 18 months of age would improve measures of cognitive and motor function in mice. Longitudinal assessment of various mouse behavior tests was completed at 20, 23, and 26 months of age to elucidate the impacts of these KD interventions on age-related decline of these functions [19–21]. Circulating markers of metabolism and inflammation, body composition, and hindlimb muscle weights were collected to provide further information on how a KD and IKD affect other common aging phenotypes.

## Methods

### Animals and diets

Male C57BL/6JN mice were obtained from the NIA Aged Rodent Colony at 11 months of age. Upon arrival, mice were group housed in polycarbonate cages on racks in a HEPA filtered room maintained on a 12-h light–dark cycle and were individually housed from 16 months of age. Health checks were performed daily. Temperature (22–24 °C) and humidity (40–60%) were controlled. Sentinel mice were housed on the same rack and given beddings from the study mice on a weekly basis. Health screens were performed on sentinel mice every 3 months and tests included aerobic cultures and serology (MHV, Sendai, PVM, MPV, MVM, M.pul and arth, TMEV [GDVII], Ectro, EDIM, MAD1 and 2, LCM, Reo-3, MNV). All tests were negative throughout the study. Mice with dermatitis were treated with weekly nail trims. All animal protocols were approved by the UC Davis Institutional Animal Care and Use Committee and were in accordance with the NIH guidelines for the Care and Use of Laboratory Animals.

When group housed, mice were provided ad libitum access to a chow diet (LabDiet 5001; LabDiet, Saint Louis, MO). At 16 months of age, the mice were individually housed and provided 11.2 kcal/day [10] of a control diet (CD). At 18 months of age, mice were randomly assigned to the control (CD), intermittent ketogenic (IKD), or ketogenic (KD) diet, with body weight counterbalanced in each group. IKD mice were fed KD meals on three consecutive days (Mon, Tue, and Wed) each week, and the KD mice were fed a KD meal each day. All mice were fed 11.2 kcal/day throughout the study. The control diet contained (% of total kcal) 10% protein, 74% carbohydrate, and 16% fat. The ketogenic diet contained 10% protein, < 0.5% carbohydrate, and 89.5% fat. Mouse diets were made in house. The CD was modified from a AIN93G diet to match the lower protein content in the KD. Table 1 provides a detailed description of the diet composition. For the CD the mineral mix TD.94046 was used, while for the KD the mineral mix TD.79055 was used instead because of its lower carbohydrate content. As TD.79055 is insufficient in calcium and phosphate, calcium phosphate and calcium carbonate supplementation was required for the KD.

**Table 1:** Experimental diets

	Control diet	Ketogenic diet
Energy density (kcal/g)	3.8	6.7
Caloric intake (kcal/day)	11.2	11.2
**Ingredients**	**g/kg diet**	
Casein	111	191
DL-methionine	1.5	2.7
Corn starch	490	–
Maltodextrin	132	–
Sucrose	100	–
Mineral mix	35	20
Vitamin mix	10	18
Soybean oil	70	70
Lard	0	582
Calcium phosphate dibasic	–	19.3
Calcium carbonate	–	8.2
Cellulose	50	85.0
TBHQ	0.014	0.126

At 27 months of age and after 9 months of dietary intervention, animals were euthanized by CO_2_ inhalation in the morning following an overnight fast. The IKD group was in the CD feeding window (after 4 days of CD) at the time of euthanasia. Tissues were collected, and wet weights of freshly dissected muscles were measured (gastrocnemius, soleus, and TA).

## Blood ketone measurement

Blood β-hydroxybutyrate level was measured using a Precision Xtra glucose and ketone monitoring system (Abbott, Chicago, IL) through tail nick.

## Body composition

Body composition was evaluated using NMR relaxometry (EchoMRI-100H, EchoMRI LLC, Houston, TX) at 27 months of age, prior to euthanasia.

## Serum analysis

Following euthanasia, blood was collected by cardiac puncture, blood samples were allowed to clot at room temperature, samples were spun at 1000 g for 10 min at 4 ˚C, and serum was sent to the UC Davis Mouse Metabolic Phenotyping Center for analysis. The following serum enzymatic colorimetric assays were completed using kits according to the manufacturer’s instructions: free fatty acids (Wako Diagnostics, Richmond, VA), triglycerides, and total cholesterol (Fisher Diagnostics, Middletown, VA). LDL and VLDL were precipitated using reagents from Abcam (Cambridge, MA), and supernatant HDL-C was measured (Fisher Diagnostics, Middletown, VA). IL-6, KC/GRO, TNF-α, and insulin were determined by electrochemiluminescent immunoassay (Meso Scale Discovery, Rockville, MD). IGF-1 and FGF-21 were determined by ELISA (R&D Systems, Minneapolis, MN).

## Hepatic lipid analysis

Snap frozen liver samples were sent to the UC Davis Mouse Metabolic Phenotyping Center for analysis. Weighed tissue samples were homogenized in methanol:chloroform (1:2). After overnight extraction, 0.7% sodium chloride was added. The aqueous layer was aspirated, and duplicate aliquots of the chloroform/lipid layer were dried under nitrogen gas. The lipid was reconstituted in isopropyl alcohol and assayed for total triglyceride (TG) and cholesterol (TC) spectrophotometrically using kits according to the manufacturer’s instructions (Fisher Diagnostics, Middletown, VA).

## Mouse behavior tests

All tests were conducted in the light cycle, and the IKD mice were fed the CD during the testing period. Testing at 20 months of age included: rearing, open field, grid wire hang, and grip strength. Testing at 23 and 26 months of age included: all the tests in the 20-month list plus the Y maze spontaneous alternation, novel object recognition, and Barnes maze.

## Open field test

The open field test was conducted in a 40 × 40 × 40 cm white acrylic box. Mice were placed at one corner of the box and allowed to freely explore the arena for 15 min. Videos were recorded with a camcorder (Sony) at an overhead view. Videos were analyzed, and motion of each mouse was tracked using the Ethovision XT15 software (Noldus, Wageningen, the Netherlands). The center zone was set as a 25 × 25 cm area in the middle of the arena. Total distance traveled and time spent in the center zone were automatically computed using the software.

## Novel object recognition test

The novel object recognition test was conducted in the same apparatus as the open field test. The 15-min open field test was used as the acclimation session for the mice to habituate to the environment. On the next day, in the morning session, two identical objects were placed in the arena, and the mouse was allowed to explore the objects for 10 min. In the afternoon session, 6 h after the morning session, one of the known objects was replaced with a novel object (the novel side was randomized among mice), and the test was performed over 10 min. A mouse was considered as exploring an object if the nose of the mouse was pointed toward the object and was within 2 cm from the object. All objects were 5–10 cm in height. The objects used for the 23-month and 26-month tests were: an orange plastic cone and a blue plastic bottle; and a small tissue culture flask filled with sand and a clear acrylic bottle filled with glass beads. Time exploring each object was manually scored using two stopwatches and % time exploring the novel object was calculated.

## Rearing test

The apparatus consisted of a clear cylinder 15 cm in diameter. Mice were placed into the cylinder and allowed to explore for 5 min. Videos were recorded and scored after the test. The number of rears in 5 min was recorded. A “rear” was defined as the mouse standing up and putting the forepaws on the side of the cylinder.

## Grid wire hang test

The apparatus consisted of a stainless-steel wire mesh screen with 1 × 1 cm grids and 1-mm wires. The screen was supported on a plastic box and was 40 cm above the surface. Towels were placed at the bottom of the box to cushion the mice. Mice were placed on the top of the screen, and the screen was gently shaken to ensure a firm grip of the mice. The screen was then inverted, and the time till the mice fell was recorded. If the maximal hanging time did not exceed 180 s, mice were given another trial after resting in the home cage for approximately 30 min (maximum of 3 trials at 20 months and 2 trials at 23 or 26 months). After 20 months of age mice did not perform better in the third trial, and the trial was deemed unnecessary at more advanced ages to prevent fatigue or additional stress. Maximal hanging impulse was calculated as (maximum hanging time (s) × body weight (kg) × 9.8 N kg^−1^).

## Grip strength

The grip strength test was performed as previously described [10] using the Imada push–pull force scale and a single metal bar (PS-500N, Northbrook, IL). Mice were given two rounds of three trials each, and the maximum grip strength (g force/body weight (g)) was used.

## Y maze spontaneous alternation test

A white acrylic Y-shaped maze was used for this test. Each arm was 120° from each other and 35 × 8 × 15 cm (L × H × W) in dimension. Mice were placed in the center of the maze, facing one arm, and allowed to move around the maze for 6 min. Videos were recorded with a camcorder at an overhead view. Motion of the mice was tracked using the Ethovision XT15 software (Noldus, Wageningen, the Netherlands). An arm entry was counted when the center point of the mouse traveled to the distal side of the arm (more than 1/3 of the arm length) and returned to the center of the maze. A non-repeating triplet is defined as when the mouse enters three different arms consecutively. The percent alternation was calculated as ((number of non-repeating triplets) ÷ (number of total arm entries − 2)) × 100%.

## Barnes maze

The maze consisted of a 92-cm plastic circular disk with twenty 5-cm holes evenly distributed on the periphery, and an overhead LED light source was used to illuminate the maze (700 lux). The maze was 75 cm above the ground. Under one of the holes, a black escape box equipped with a step and filled with fresh bedding was attached to serve as a shelter for the mice. Signs were placed around the maze to be used as visual cues. During the training trials, mice were placed in the middle of the maze under an inverted opaque bucket. After 10 s, the light was turned on, and the bucket was immediately lifted. Mice were allowed to explore the maze for 3 min or until they entered the escape box through the target hole. If the mice did not enter the target hole within 3 min, the mice were gently directed to the target hole. Mice were trained for 3 trials a day for 3 days, and the intertrial interval was 15–20 min. On the probe day, the escape box was removed, and the trial lasted for 2 min. Mice that did not find the target hole in 2 min in the probe trial were considered as outliers and were excluded from the analysis. Videos were recorded using an overhead GigE camera connected to a computer and analyzed using the Ethovision XT15 software (Noldus, Wageningen, the Netherlands). Latency to the target hole and time spent in the target quadrant (quarter of the maze with the target hole in the middle) were calculated automatically using the software.

## Statistical analysis

All values are expressed as mean ± SEM, and *p* values < 0.05 were considered significant. All analyses were performed using GraphPad Prism 9.3 (GraphPad Software Inc., San Diego, CA). Outliers within each group were detected using the Grubbs’ test. Body weight, circulating BHB, and behavior data were analyzed using repeated measures two-way analysis of variances (ANOVA), followed with the Tukey’s or Bonferroni post-hoc tests for pairwise group comparisons, to compare differences between diets and time points (ages) respectively. Cross-sectional comparisons between diets in other measurements were analyzed using ordinary one-way ANOVAs with the Tukey’s post hoc tests or the Brown-Forsythe and Welch ANOVAs with the Dunnett’s T3 post hoc tests. When needed, log transformation was applied to data to approximately conform to normality prior to one-way ANOVAs. The *z*-score for each behavior test was calculated as the number of standard deviations from the mean of the reference group (20-month CD group for the motor function score and 23-month CD for the cognition score), and a composite score was then computed by averaging the z-scores from all the tests. The motor function score included results from the grid wire hang, grip strength, distance traveled in the open field, and rearing count. The cognition score included % time exploring the novel object in the novel object recognition test, % alternation in the Y maze, latency to the target hole in the Barnes maze probe trial, and time spent in the target quadrant in the Barnes maze probe trial. Mice that were outliers in any behavior tests were excluded from the computation of the composite score. The directionality of scores were adjusted, and a higher positive score indicated better performance.

## Results

### KDs started in late-middle-aged mice distinctively altered body weight and blood ketone level

**Fig. 1:**
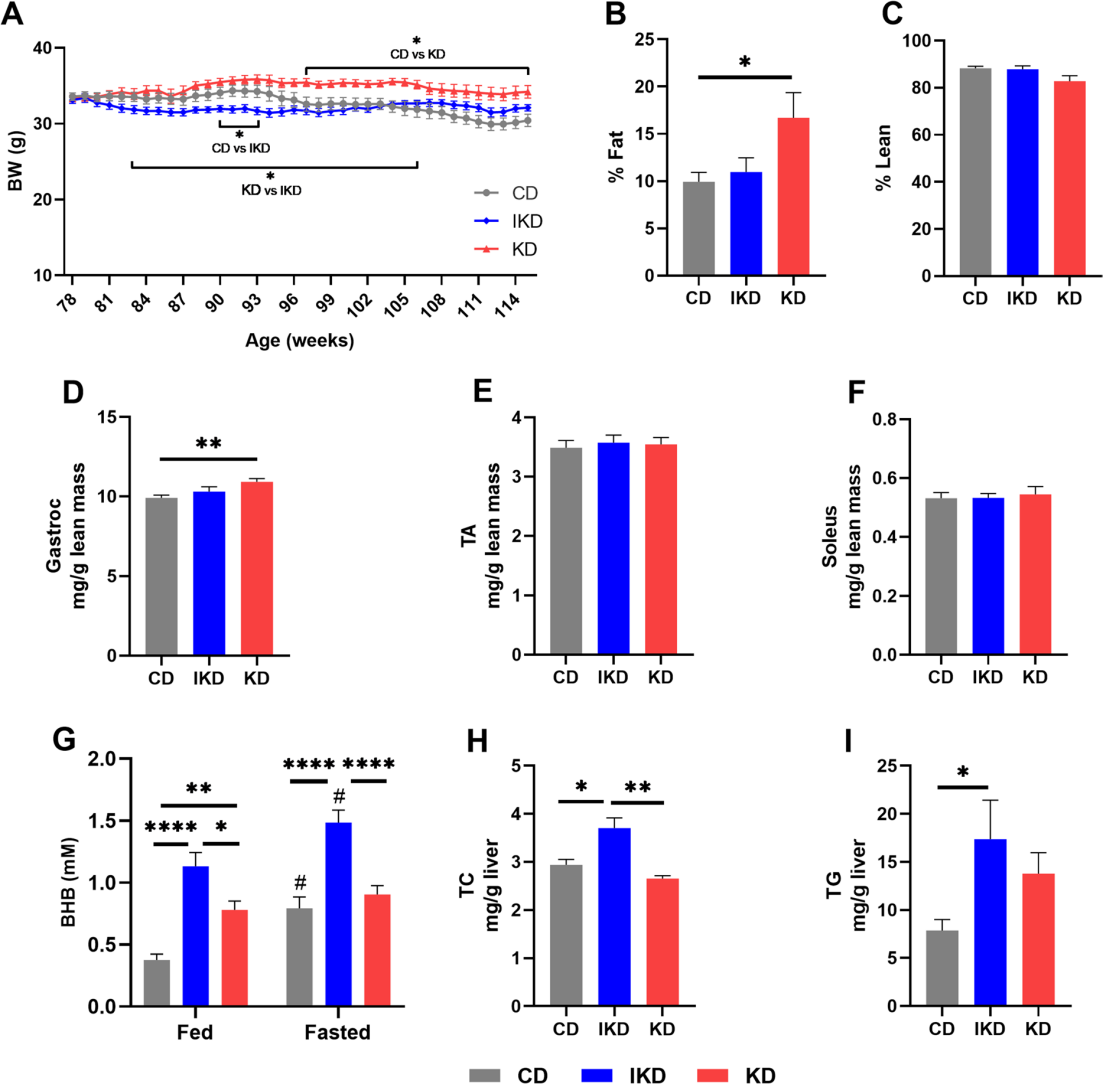
**Differential adaptation of 18-month male mice to a KD or IKD**. (**A**) Body weights of CD, IKD, and KD mice throughout the study (*n* = 19–20/group). (**B**–**C**) Percent fat (**B**) and lean (**C**) mass measured using EchoMRI at 27 months of age (*n* = 10–12/group). (**D**–**F**) Relative mass of gastrocnemius (**D**), tibialis anterior (**E**), and soleus (**F**) muscle to total lean mass (*n* = 10–12/group). (**G**) Circulating level of β-hydroxybutyrate (*n* = 19–20/group). (**H**–**I**) Content of liver total cholesterol (**H**) and triglyceride (**I**) (*n* = 8/group). Diets: CD—control, IKD—intermittent ketogenic, KD—ketogenic. ∗ *p* < 0.05, ∗  ∗ *p* < 0.01, ∗  ∗  ∗  ∗ *p* < 0.0001, #*p* < 0.05 between fasted vs. fed ketone levels, based on repeated measures two-way ANOVA or one-way ANOVA followed with Tukey’s multiple comparisons tests

Although all groups were fed an isocaloric amount of diet, KD mice were heavier than the CD mice after 19 weeks on diet (Fig. 1A) and maintained this weight throughout the study. IKD mice had an initial drop in body weight, which was significantly lower than the CD mice at 12–16 weeks on diet, but then they maintained weight with aging and were no longer different than CD (Fig. 1A). Body composition measurements at 27 months of age, or after 9 months of intervention, showed that KD mice had a higher fat mass (Fig. 1B) compared to CD mice, while lean mass (Fig. 1C) was not different among diet groups, consistent with the increase in KD body weight being mostly body fat. Weights of representative muscles from the lower hindlimb were also measured to assess preservation of muscle mass at advanced age. KD mice had relatively larger gastrocnemius muscles (Fig. 1D) compared to CD mice, while weights of tibialis anterior and soleus were not different among groups (Fig. 1E–F).

Circulating β-hydroxybutyrate was measured to assess the level of ketogenesis with consumption of a KD. Both KD and IKD mice had an elevated 3-h postprandial, and IKD mice had a higher fasted blood β-hydroxybutyrate level compared to the CD mice (Fig. 1G). IKD mice also had significantly higher blood β-hydroxybutyrate than KD mice in either the fed or fasted states (Fig. 1G).

To evaluate the effects of KDs on liver lipid content, triglyceride and cholesterol levels were measured. There was no difference in liver triglyceride or cholesterol levels between KD and CD mice, while the IKD mice had an elevated cholesterol content compared to both the CD and KD mice and an increased triglyceride content compared to CD mice (Fig. 1H, I).

### Circulating proinflammatory cytokines were decreased, and cholesterol levels were differentially altered with KDs

**Fig. 2:**
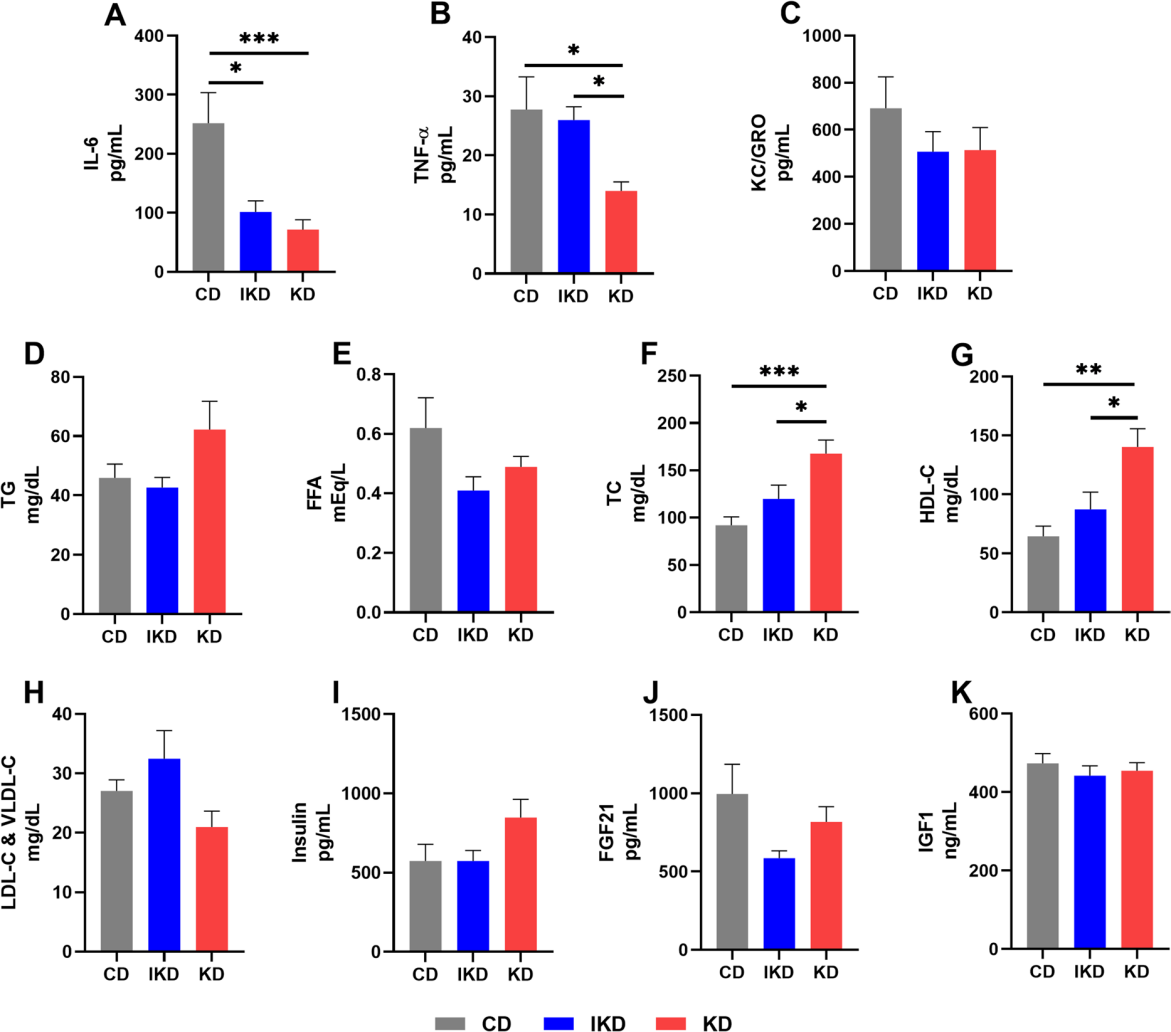
**Levels of circulating proinflammatory cytokines and markers of metabolism at 27 months of age in male mice**. Circulating levels of (**A**) IL-6, (**B**) TNF-α, (**C**) KC/GRO, (**D**) total triglyceride (TG), (**E**) free fatty acids (FFA), (**F**) total cholesterol (TC), (**G**) HDL-C, (**H**) LDL-C and VLDL-C, (**I**) insulin, (**J**) fibroblast growth factor-21 (FGF21), and (**K**) insulin like growth factor-1 (IGF1) (*n* = 10/group). Diets: CD—control, IKD—intermittent ketogenic, KD—ketogenic. ∗ *p* < 0.05, ∗  ∗ *p* < 0.01, ∗  ∗  ∗ *p* < 0.001 based on one-way ANOVA followed with the Tukey’s multiple comparisons test or the Brown-Forsythe and Welch ANOVAs followed with the Dunnett’s T3 multiple comparisons test where appropriate

To investigate changes in inflammation and metabolism with the KDs, a panel of serum markers were measured. Serum IL-6 was significantly decreased in both KD and IKD groups at 27 months of age compared to CD (Fig. 2A). KD mice also had a significantly lower level of TNF-α compared to both CD and IKD mice (Fig. 2B). KC/GRO was not different among diet groups (Fig. 2C).

Serum triglycerides (TG) and free fatty acids (FFA) were not different among diet groups (Fig. 2D, E). Serum total cholesterol as well as HDL-C levels were elevated in the KD group (Fig. 2F, G) compared to CD and IKD group. Levels of serum LDL-C and VLDL-C were not increased with the KD (Fig. 2H), indicating the elevation in total cholesterol was largely due to the increase in HDL-C. In fact, KD mice showed a trend (*p* = 0.06) toward decreased LDL-C and VLDL-C compared to the IKD mice (Fig. 2H). Circulating levels of hormones that regulate metabolic pathways were also measured. Insulin, fibroblast growth factor 21 (FGF21), and Insulin like growth factor 1 (IGF1) (Fig. 2I–K) were not significantly altered by any of the dietary interventions in the fasted state.

### Age-related declines in measures of motor function were observed in all groups, and diet-associated improvement in grid wire hang was observed with KD and IKD

**Fig. 3:**
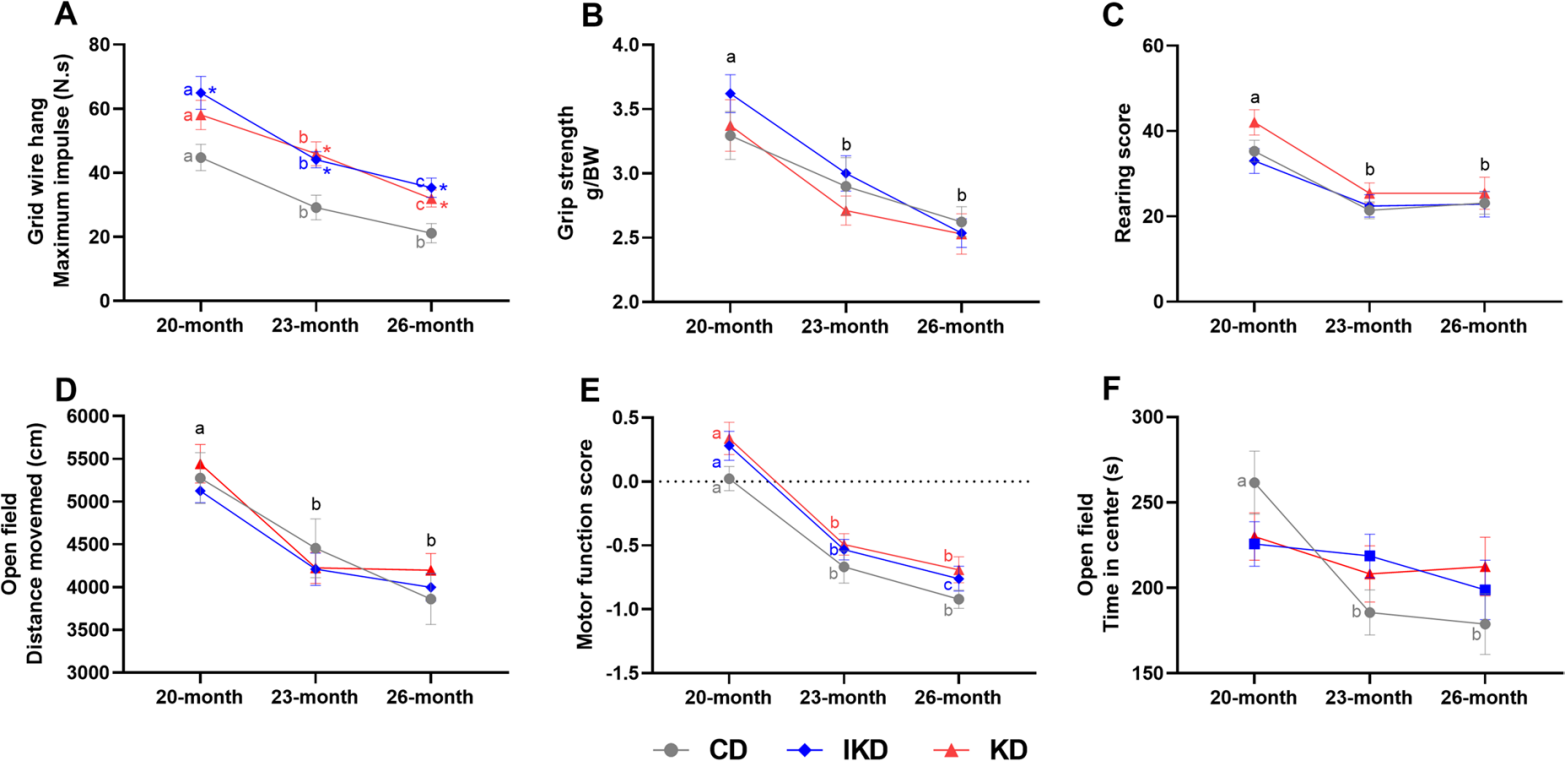
**Behavior tests of motor function and anxiety in male mice at 20, 23, and 26 months of age**. (**A**) Grid wire hang test: maximum hanging impulse. (**B**) Grip strength test: relative peak force of forelimbs exerted on the force meter. (**C**) Rearing score: number of wall-contact rears by the front paws of the animal. (**D**) Open field test: total distance moved as a measure of locomotor activity. (**E**) Composite score of motor function tests (data from **A**–**D**). (**F**) Open field test: time spent in the center region as a measure of anxiety (not included in the composite score of motor function tests). 20 months: *n* = 18–20; 23 months: *n* = 17–18; 26 months: *n* = 14–17/group. Diets: CD—control, IKD—intermittent ketogenic, KD—ketogenic. Data were analyzed by repeated measures two-way ANOVA followed with the Tukey’s or the Bonferroni multiple comparisons tests. ∗ *p* < 0.05 compared to the CD group at the same age. Different letters of a, b, or c denote difference (*p* < 0.05) between ages

Muscle strength and endurance under isometric contraction were assessed using the grip strength and grid wire hang tests. Mice fed a KD or IKD were more resistant to falling from the grid wire at 20 (KD showed a trend although not significant, *p* = 0.09), 23, and 26 months of age (Fig. 3A). Grip strength performance was not different among diet groups at any age (Fig. 3B). Locomotor activity was evaluated using the rearing and open field tests. Neither rearing score nor total distance traveled in the open field was altered with KD or IKD at any age (Fig. 3C, D).

Age-related declines in grid wire hang maximum impulse, grip strength, rearing score, and total distance traveled in open field were observed after 20 months of age in all diet groups (Fig. 3A–D). A composite score consisting of the parameters in all the motor function tests was computed to give an indication of motor performance (Fig. 3E). As expected, age-related decline in motor function score after 20 months of age was observed in all diet groups, and the score did not differ among diets. Time spent in the center of the open field was decreased in the CD mice after 20 months of age (Fig. 3F), consistent with an age-related increase in anxiety level, and such a change was not observed in IKD or KD mice with aging.

### KD and IKD showed improvements in spatial memory at old age

**Fig. 4:**
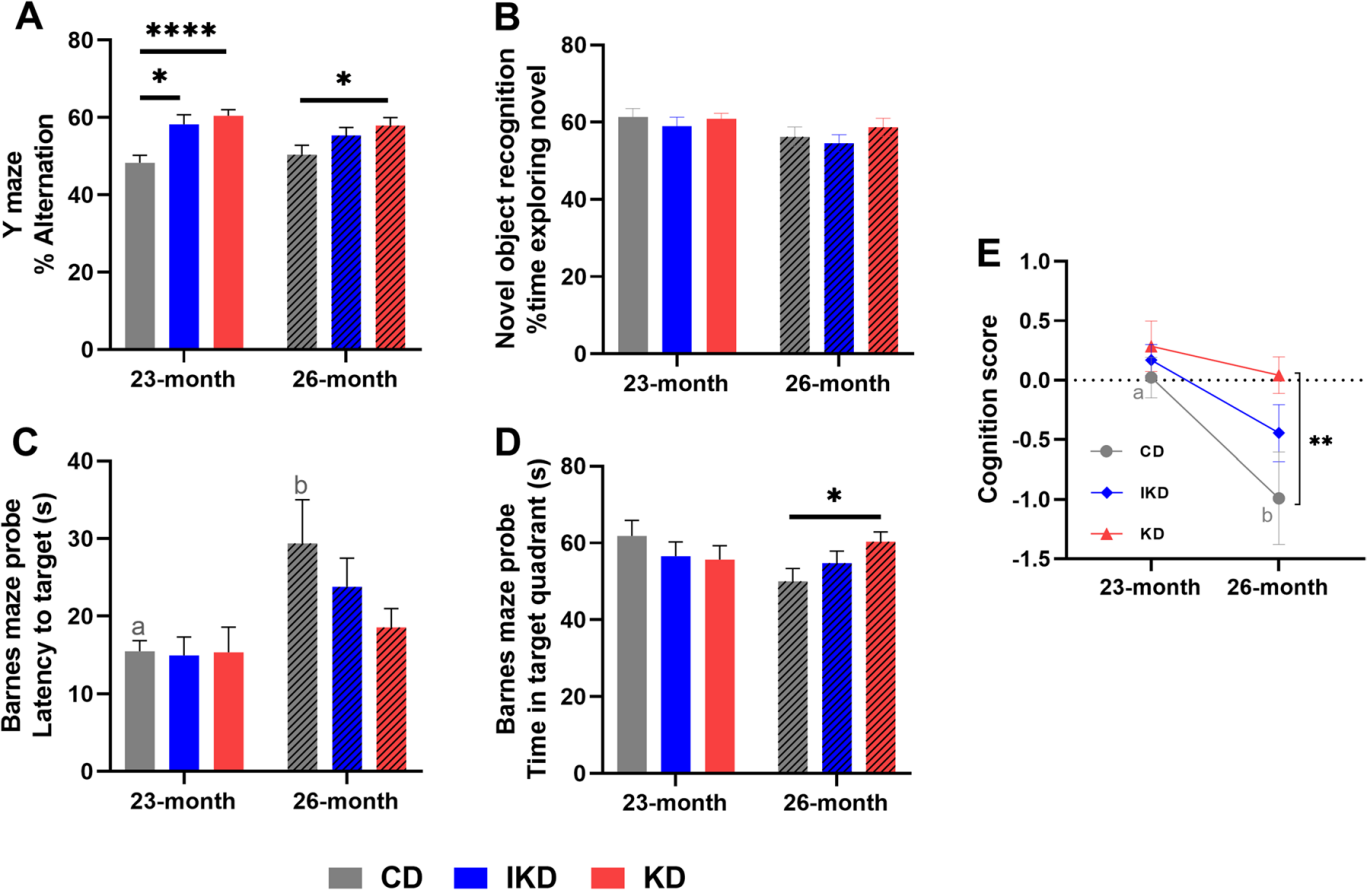
**Behavior tests of cognitive function in male mice at 23 and 26 months of age**. (**A**) Y maze spontaneous alternation test: percent of alternations. (**B**) Novel object test: percent time exploring the novel object. (**C**) Barnes maze test: latency to the target hole in the probe trial (lower the better). (**D**) Barnes maze test: time spent in the target quadrant in the probe trial (higher the better). (**E**) Composite of cognitive tests. 23 months: *n* = 17–18; 26 months: *n* = 14–17/group. Diets: CD—control, IKD—intermittent ketogenic, KD—ketogenic. Data were analyzed by repeated measures two-way ANOVA followed with the Tukey’s or the Bonferroni multiple comparisons tests. ∗ *p* < 0.05, ∗  ∗  ∗  ∗ *p* < 0.0001. Different letters of a and b denote difference (*p* < 0.05) between ages

At 23 months of age, both KD and IKD mice showed a higher percent of alternating triplets in the Y maze spontaneous alternation test (Fig. 4A), consistent with an improved spatial working memory. At 26 months of age, KD mice still performed significantly better in Y maze compared to CD mice, while the performance of IKD mice was not significantly different from CD or KD mice but intermediate between CD and KD at this age.

Object recognition memory, assessed using the novel object test, was not altered with any diets (Fig. 4B) at any age.

Improved spatial learning memory, which was assessed using the Barnes maze, was observed for the KD mice at 26 months of age (Fig. 4C–D), represented as a significant increase in time spent in the target quadrant compared to the CD group. Spatial learning memory was not altered at 23 months of age among any diet groups. A significant age-related increase in latency and a trend (*p* = 0.06) of decreased time spent in target quadrant were detected in CD, but not IKD or KD mice, consistent with preservation of spatial learning memory in the IKD and KD groups at advanced age.

To evaluate overall effects of age or diet on cognition, parameters of the cognitive tests were combined to calculate a composite cognition score normalized to the mean performance of 23-month CD mice (Fig. 4E). At 26 months of age, KD mice had a higher cognition score compared to CD mice with IKD mice showing a cognition score intermediate to KD and CD. CD mice showed a decrease in cognition score from 23 to 26 months of age, while both the IKD and KD mice showed no significant declines with aging.

Overall, these results suggest that a KD improved some measures of cognition at old age compared to CD mice. IKD mice improved spatial working memory compared to CD mice at 23 months of age, and at 26 months of age showed measures of memory intermediate to KD and CD mice. Both IKD and KD did not have significant decreases in measures of memory at advanced age, while an age-related difference was observed with the CD.

## Discussion

The beneficial effects of KDs on longevity and health span have been studied in early-middle-aged (12-month-old) mice [10, 18]. However, it is not known if KDs initiated at late middle-age would confer similar effects. In addition, a KD administrated intermittently for a brief period is also of particular interest as a strategy to mimic changes that occur with intermittent fasting. An intermittent metabolic switch between glucose and ketone body utilization has been proposed to optimize cognitive functions in aging [22], and mice cycled on a weekly KD had reduced mortality and enhanced health span [18]. The main goal of this study was to induce continuous or intermittent ketosis (3 days per week) in late-middle-aged mice with KDs and assess whether these dietary interventions improve cognitive and motor functions at old ages. Our results demonstrate that a KD started at 18 months of age improved performance in some measures of cognitive and muscle function at 26 months of age, and a weekly 3-day IKD showed effects intermediate to KD and CD. Also, tests were carried out when the IKD mice were on a CD, and this might be particularly important as calorie restriction or intermittent fasting does not produce continuous ketosis.

Aging is associated with a gradual decline in cognitive function, and the pro cognition effects of KDs have been demonstrated in some studies using aged mice [10, 18]. Declines in spatial learning and working memory with aging have been shown [23, 24], and our results demonstrate that these memory functions were improved in mice fed a KD compared to CD at 26 months of age, and mice fed an IKD had improved spatial working memory at 23 months of age. Although previous studies showed that KDs started in 12-month old mice improved recognition memory in the novel object recognition test [10, 18], performance in the novel object test was not altered with KD or IKD in this study, and this suggests that KDs initiated at early middle age may be necessary to render an improvement in recognition memory when fed isocalorically to a CD. Regional differences in KD’s effect on brain may explain the observation that certain memory tasks were improved while others were not. Spatial memory is more susceptible to neurodegeneration in the hippocampus, and recognition memory is impacted to a much lesser extent [25]. Studies have also shown that a KD differentially alters synaptic morphology in rat hippocampus [26]. Moreover, it has been reported that in old mice calorie restriction improved spatial leaning memory evaluated through the Barnes maze but not recognition memory measured with the novel object test [27], consistent with our findings that memory tasks can be variably impacted by diet. Nevertheless, results of the present study highlight the potential therapeutic effects of KDs on attenuating age-related decline in spatial learning and working memory. The mechanisms responsible for KD-related changes in cognitive function are not entirely known. Several mechanisms associated with the effects of KDs on cognition in aged animals have been proposed, including an elevation in acetylation level of hippocampal histone proteins [3, 28], the attenuation of age-related decline in synaptic plasticity [29], an upregulation in BDNF signaling [22], and an increase in mitochondrial abundance in some neuronal populations[30]. Additional work is needed to determine if any of these mechanisms contribute to the cognitive changes observed in the present study.

A decline in motor function and muscle strength also occurs with aging [20], and our results showed that locomotor activity in a novel environment, grip strength, and wire hang performance were reduced with age in all diet groups. Although age-related decline in these measures of motor functions was not prevented with a KD or IKD, a significant improvement in grid wire hang performance was observed in KD and IKD mice. This suggests that muscle endurance under isometric contraction measured with the grid wire hang test was more profoundly affected by KDs when started in late middle age, while grip strength, a measure of maximal isometric strength of the forelimb only, was not significantly impacted. Low carbohydrate diets have gained popularity with endurance athletes, and studies have reported that KDs and KEs may improve performance in some endurance sports [31]. Our findings were consistent with the notion that KDs may improve or preserve muscle isometric contraction endurance with age. Recent studies have demonstrated KD started in middle age preserved hindlimb muscle mass better than the control mice at advanced age [10, 32], and a ketone ester (KE) mitigated gastrocnemius, although not soleus or TA, muscle loss in a mouse model of muscle atrophy [33]. Our observation that the gastrocnemius weight was increased in the KD mice at advanced age is consistent with these findings.

Our results also show that CD mice spent less time in the center region in the open field test at 23 and 26 months of age, while that of KD and IKD mice was not impacted by aging, consistent with previous studies that reported anxiolytic effects of KDs in laboratory rodents [34].

Age-related diseases are associated with chronic inflammation and accompanied by an elevation in proinflammatory cytokines. Our results support the notion that a metabolic shift from glucose to ketone body utilization decreased level of inflammation [22]. Of particular interest, the level of serum IL-6 was significantly decreased in both aged IKD and KD mice, and a lower level of IL-6 has been associated with a reduced risk of experiencing cognitive decline with aging [35, 36]. Moreover, increased circulating levels of IL-6 can influence skeletal muscle redox balance [37] and is associated with a decline in muscle strength and function [38]. A reduced level of circulating IL-6 in KD and IKD mice is consistent with our results that cognition and wire hang performance were improved in aged mouse. To study the effects of KDs started in late middle age on metabolism, a panel of circulating metabolites and hormones were measured. KD started at 18 months of age increased total circulating cholesterol, and this change was not observed with a KD started at 12 months of age [10]. The increase in circulating cholesterol in the KD mice was primarily due to an increase in HDL-C, which is associated with anti-inflammatory effects and enhanced endothelial wall function [39]. Although a significant increase in liver triglyceride and cholesterol were detected in IKD mice, the levels were still within the range of normal aged C57BL/6 mice [40, 41] and were far below the level of high fat fed aged animals [42, 43]. The mild elevation in liver triglyceride and cholesterol may be an indication that the IKD mice were going through shifts in metabolism under the dramatic, cyclical changes induced by their feeding strategy. After the IKD mice were refed the high-carbohydrate CD, the lipogenesis pathways in liver may be upregulated, as seen in animals during the refeeding phase of an intermittent fasting regime [44], and at the end of the CD phase (time of euthanasia) higher level of triglyceride was accumulated. On the other hand, when the IKD mice were in the KD phase, a significantly higher circulating β-hydroxybutyrate level was observed in both fasted and fed states, suggesting either upregulated ketogenesis or reduced ketone uptake by peripheral tissues. However, future studies are needed to determine the specific metabolic changes that occur with intermittent KD approaches.

We recognize limitations to the present study. C57BL/6J mice show heterogeneity in aging and age-associated functional decline [11], and baseline motor and cognitive function measurements would be useful in determining age-related changes in these functions for each diet group. Diet assignment based on baseline measurements of motor and cognitive functions at late middle-age would ensure that mice with various levels of performance were evenly distributed in different dietary interventions. Since spatial working memory was improved at 23 months of age with both IKD and KD but Y maze was not conducted at a younger age, it is possible that these interventions would improve Y maze performance at 20 months of age, and it is important to determine how quickly spatial working memory is altered with KD approaches. Furthermore, GTT or ITT was not performed, which would be useful to show the metabolic adaptations with intermittent and continuous KD started at late middle age. Finally, a female cohort was not included in the present study, and we recognize that more work needs to be done to investigate the effect of KDs on middle-aged and aged female mice.

Overall, our results demonstrate that KDs started in late middle-age are effective at improving spatial memory and wire hang performance. A KD had the most potent effect in aged mice with IKD showing results intermediate to the KD and CD diet groups.

## Data Availability

The data used to form the figures can be provided by the authors on request.
